# Study on the Multitarget Mechanism and Active Compounds of Essential Oil from *Artemisia argyi* Treating Pressure Injuries Based on Network Pharmacology

**DOI:** 10.1155/2022/1019289

**Published:** 2022-01-19

**Authors:** Shu-ting Lu, Lu-lu Tang, Ling-han Zhou, Ying-tao Lai, Lan-xing Liu, Yifan Duan

**Affiliations:** ^1^The First College of Clinical Medicine, Guangzhou University of Chinese Medicine, Guangzhou, China; ^2^Department of Anesthesiology, The First Affiliated Hospital, Guangzhou University of Chinese Medicine, Guangzhou, China

## Abstract

In order to comprehensively explore multitarget mechanism and key active compounds of *Artemisia argyi* essential oil (AAEO) in the treatment of pressure injuries (PIs), we analyzed the biological functions and pathways involved in the intersection targets of AAEO and PIs based on network pharmacology, and the affinity of AAEO active compounds and core targets was verified by molecular docking finally. In our study, we first screened 54 effective components according to the relative content and biological activity. In total, 103 targets related to active compounds of AAEO and 2760 targets associated with PIs were obtained, respectively, and 50 key targets were overlapped by Venny 2.1.0. The construction of key targets-compounds network was achieved by the STRING database and Cytoscape 3.7.2 software. GO analysis from Matespace shows that GO results are mainly enriched in biological processes, including adrenergic receptor activity, neurotransmitter clearance, and neurotransmitter metabolic process. KEGG analysis by the David and Kobas website shows that the key targets can achieve the treatment on PIs through a pathway in cancer, PI3K-Akt signaling pathway, human immunodeficiency virus 1 infection, MAPK signaling pathway, Wnt signaling pathway, etc. In addition, molecular docking results from the CB-Dock server indicated that active compounds of AAEO had good activity docking with the first 10 key targets. In conclusion, the potential targets and regulatory molecular mechanisms of AAEO in the treatment of PIs were analyzed by network pharmacology and molecular docking. AAEO can cure PIs through the synergistic effect of multicomponent, multitarget, and multipathway, providing a theoretical basis and new direction for further study.

## 1. Introduction

Pressure injuries (PIs), also named pressure ulcers, refer to localized injuries occurring in the skin and/or potential subcutaneous soft tissue, usually occurring in bone bulges or in contact with medical facilities [1]. PIs have the characteristics of refractory, high incidence, and high treatment cost [2, 3]. Once infected, it is easy to cause sepsis and death [4]. At present, the treatment of PIs mainly includes drug therapy [5], dressing therapy [6], stem cell factor therapy [7], and negative pressure wound therapy [8]. There are no effective measures yet; expert consensus believes that prevention and early treatment are crucial [9].

The *Artemisia argyi* (AA), which is widely distributed in China and other Asian countries, has been used as traditional medicine or food supplement for hundreds of years [10]. AA is the dried leaf of *Artemisia argyi* (Levl.) et Van., the herb with a spicy, bitter flavor and warm properties, enters into the channels of liver and kidney, and functions on resolving blood stasis, dispersing cold and relieving pain [11, 12]. AA is rich in volatile essential oils (AAEO), such as eucalyptol, camphor, and borneol, with extensive pharmacological effects of antioxidative stress [13], resisting pathogens [14], suppressing inflammatory responses [15], and activating immunomodulatory responses [16].

AA often treats diseases in the form of moxibustion; moxibustion is a critical intervention in traditional Chinese medicine (TCM). *Artemisia argyi* is usually the main raw material [17]. Although the mechanism of moxibustion is uncertain, the thermal effect and moxa smoke may play a synergistic role in the treatment of diseases [18, 19]. The fumigation and heating effects produced by moxibustion have played a certain role in promoting the wound healing of PIs, and the pharmacological effects of moxa smoke need to be paid special attention. Nevertheless, we found that moxa smoke and AAEO have 80% of the same compounds by searching the relevant literature. Also, in view of the increasing emphasis on the toxicity of moxa smoke to cardiovascular and respiratory systems, AAEO is safer.

In view of the complex chemical compounds of AAEO, the chemical components and the corresponding mechanism of action that play the efficacy after entering the human body include a lot of unknown information. Therefore, it is necessary to comprehensively explore the mechanism of AAEO in the treatment of PIs.

Network pharmacology is a new discipline emerging in recent years that combines the overall network analysis and pharmacological effects [20]. With the development of bioinformatics and chemical informatics, network pharmacology has become a new method to study the mechanism of traditional drugs and discover potential bioactive components effectively and systematically [21]. Network pharmacology explores the relationship between drugs and diseases from a holistic perspective and, through a large number of databases screening drug treatment of diseases related targets and pathways, is widely used in TCM-related fields, providing new ideas for the study of complex Chinese medicine system [20, 22, 23]. Molecular docking, as a new technology for drug molecular screening, utilizes one-to-one pairs of ligands and receptors according to the “lock-key principle,” the computer-aided high-throughput screening of drug molecules was realized by studying the geometric matching and energy matching between protein macromolecular receptors and small drug molecules, and the mechanism of drug molecules was further predicted to improve the scientificity, accuracy, sensitivity, and predictability of drug molecule screening [24].

For all we know, our study is first time applied network pharmacology methods to explore the biological effect of active compounds in AAEO and the multitarget mechanism of active compounds in the treatment of PIs. In our study, TNF, PTGS2, IL6, IL1*β*, NR3C1, CASP3, TP53, PGR, REN, and NOS2 could be the potential receptor targets, involving many inflammatory proteins. The top three molecular docking points are PTGS2 (prostaglandin-endoperoxide synthase 2), TP53 (tumor protein p53), and PGR (progesterone receptor). PTGS2, also known as COX-2, as an important inflammatory mediator, exists in the early stage of inflammation to the whole process of inflammation formation [25]. It is upregulated when stimulated by various stimuli and participates in various pathological processes, closely related to inflammation, tumor occurrence, and development [26, 27]. TP53 and PGR are tumor suppressor proteins, being a biomarker and prognostic predictor of cancers usually [28–31]. Recent studies have shown that TP53 plays an important role in regulating signaling pathways to maintain the health and function of skeletal muscle cells. It can improve cell survival rate by participating in the activation to increase the repair time of cells and prevent abnormal cell proliferation through the initiation of DNA fragmentation-induced apoptosis to promote the increase of cell stress level [32].

## 2. Methods

### 2.1. Active Compounds of AAEO Database Building and Screening

Over 200 components of AAEO can be detected by current technology, but more than 90 of them are common active, so we use 94 components as active compounds [33, 34]. Fifty-four compounds were screened by criteria. Finally, the inclusion criteria were as follows: the compounds with relative content >0.1% from works of literature of GS-MC quantitative analysis (hydrodistillation) of AAEO in recent years [14, 35, 36], compounds included in TCMSP [37] (https://tcmspw.com) and PubChem database [38] (https://pubchem.ncbi.nlm.nih.gov/), and compounds with relevant targets.

### 2.2. Targets Fishing

The targets information identifying 54 potential compounds were attained on TCMSP and were reconfirmed by DrugBank [39] (https://www.drugbank.ca) and Pharmmapper [40] (https://www.lilab-ecust.cn/pharmmapper/). Next, the targets were entered into UniProt (https://www.uniprot.org/); the species selected was “Homo sapiens”; transformed gene symbols were obtained finally.

GeneCard (https://www.genecards.org/), OMIM (https://omim.org/) and DrugBank (https://go.drugbank.com/) database were used to screen relative targets of PIs. “Pressure Ulcers,” “Bedsore,” “Pressure Sore,” and “pressure injury” were keywords to search targets related to PIs. The obtained targets were integrated and eliminated duplication. Finally, the intersection targets were obtained on Venny 2.1.0 (https://bioinfogp.cnb.csic.es/tools/venny/). At last, 50 overlapping targets were obtained.

### 2.3. PPI Analysis and Compounds-Targets Network Construction

PPI analysis of the overlapping targets was carried out in the STRING 11.0 (https://www.string-db.org/). Protein with disconnected other protein and a combined score <0.4 was removed [41]. The information of the PPI network was visualized by Cytoscape 3.7.2 software [42]; then, core network calculations were performed by the Cytoscape plug-in module, MCODE, the degree of freedom threshold was set as 100, the node scoring threshold was 0.2, the *K* value was 2, and the maximum depth was 100 [43].

### 2.4. Gene Ontology (GO) Analysis

The overlapping targets were imported into Matescape [44] (https://metascape.org/gp/index.html) to carry out GO analysis. The specific steps were as follows: input the gene ID, the parameter selected was “Homo sapiens,” click “custom analysis,” and click GO Molecular Functions, GO Biological Processes, and GO Cellular Components in turn for analysis [44]. Finally, Bioinformatics (https://www.bioinformatics.com.cn/) was used to acquire the visualization of the results.

### 2.5. Kyoto Encyclopedia of Genes and Genomes (KEGG) Pathways Analysis

50 overlapping targets were converted from gene symbol to ENTRZ_GENE ID in David Database (https://david.ncifcrf.gov/tools.jsp), and the ENTRZ_GENE ID was input into Kobas (https://kobas.cbi.pku.edu.cn/) for KEGG pathways analysis [45, 46]. KEGG pathways with *P* values <0.01 were selected [47].

### 2.6. Molecular Docking

In silico methods are alternatives to experimental approaches to screen for potential bioactivity of compounds of essential oil compounds; for example, docking evaluated in silico the ability of EOs to interact with molecular targets with advantages of being less time-consuming and cheap. We selected the top 10 core targets and got the ligand with relative content of the first 7 for molecular docking; the PDB formats of proteins were obtained from the protein database (https://www.rcsb.org) and ligand files in mol2 formats from PubChem (https://pubchem.ncbi.nlm.nih.gov/) [48]; both of them were used in the same way they were obtained from the databases. Molecular docking was carried out in CB-Dock (https://cao.labshare.cn/cb-dock/). CB-Dock server is a user-friendly blind docking network server developed by Dr. Liu's research team. It uses a novel curvature-based cavity detection approach, and Autodock Vina, the popular docking program, is used for docking [49]. The success rate of this tool was more than 70%, which outperformed the state-of-the-art blind docking tools. The downloaded formats files were input into CB-Dock; the style and color of ligand and receptor were set the same as those of Dr. Tao [50]. The RMSD between each pair of the two structures must be less than 2 angstroms [51].

## 3. Results

### 3.1. Compounds of AAEO and Targets Related to Active Compounds

A total of 54 active compounds that met the criteria were finally collected. The basic information of 54 obtained compounds is shown in Table 1.

### 3.2. Targets' Intersection and PPI Network Construction

**Figure 1:**
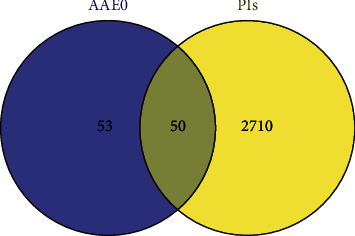
Venn diagram of targets' intersection of AAEO and PIs.

**Figure 2:**
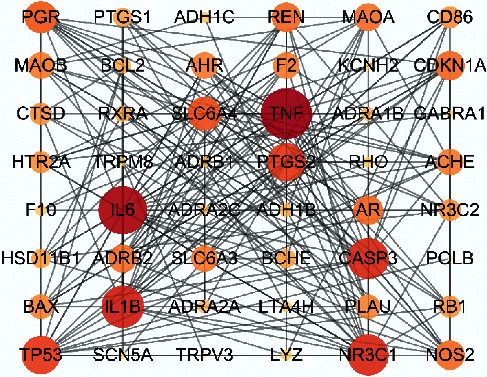
PPI network diagram. Protein-protein interactions (*P* > 0.7) of 50 overlapping targets.

103 AAEO compound-related targets were retrieved from TCMSP and converted into official gene symbols according to the UniProt database. Moreover, 2760 PIs targets were searched by GeneCard, OMIM, and DrugBank databases. Finally, 50 targets were obtained by intersecting two parts of targets (Figure 1); the PPIs of 50 overlapping targets are shown in Figure 2.

### 3.3. Active Compounds and Overlapping Targets Network Construction

**Figure 3:**
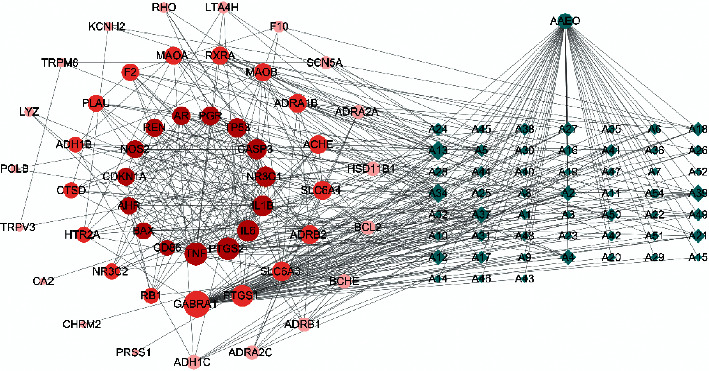
Compounds-overlapping targets network: the right square matrix green circle nodes represent 52 potential compounds (2 compounds (A33 and A53) have no associated targets) and the left circular nodes with gradual color represent 50 overlapping targets of AAEO and PIs. Larger size and deeper color of a node mean a greater degree.

Compounds-overlapping targets network involved 104 nodes and 441 edges. The results reflect the complex mechanism of multicomponent and multitarget treatment of diseases. Moreover, a core network was calculated by MCODE with 15 targets (Figure 3).

### 3.4. GO Analysis of Targets' Intersection

**Figure 4:**
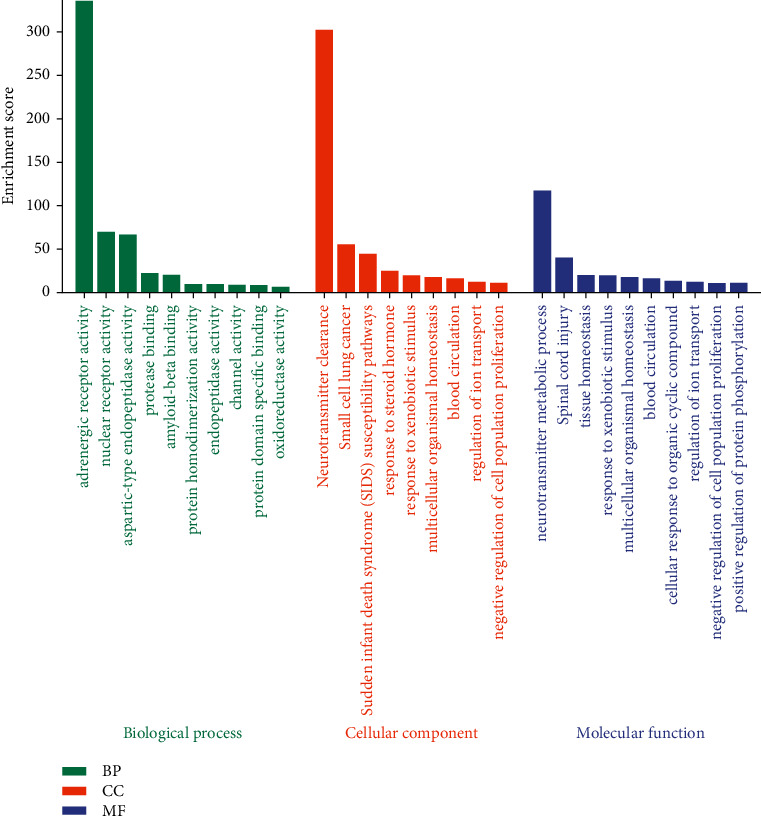
GO analysis.

GO analysis was mainly focused on the biological process, with a total of 3269 enrichment results, involving adrenergic receptor activity, nuclear receptor activity, and aspartic-type endopeptidase activity. The top 10 GO functional annotations of BP, CC, and MF are shown in Figure 4.

The top 10 GO functional annotations of BP, CC, and MF are represented by green for biological process, orange for cellular component, light purple for molecular function, respectively.

### 3.5. KEGG Pathways of Targets' Intersection

**Figure 5:**
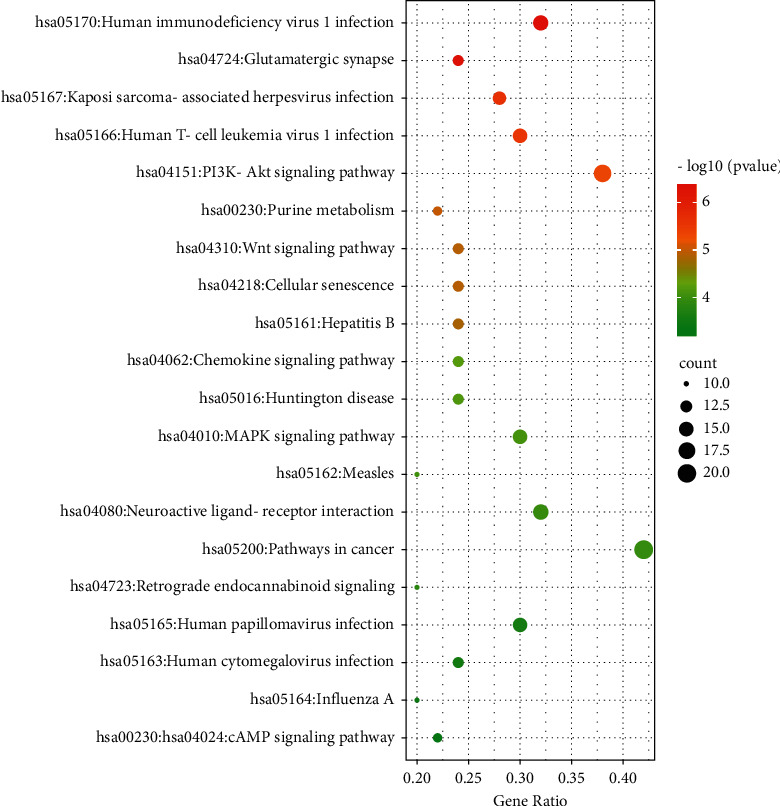
Top 20 enriched KEGG pathways.

KEGG enrichment results were involved in 128 pathways, including pathway in cancer, PI3K-Akt signaling pathway, human immunodeficiency virus 1 infection, MAPK signaling pathway, and Wnt signaling pathway. The top 20 pathways were selected by cluster analysis and *P*-value (Figure 5).

Each bubble represents an enriched function, and the size of the bubble is from small to large. The bubble is colored according to its −log (*P* value); when the color is redder, *P* value is smaller.

### 3.6. Compound-Target Docking

**Figure 6:**
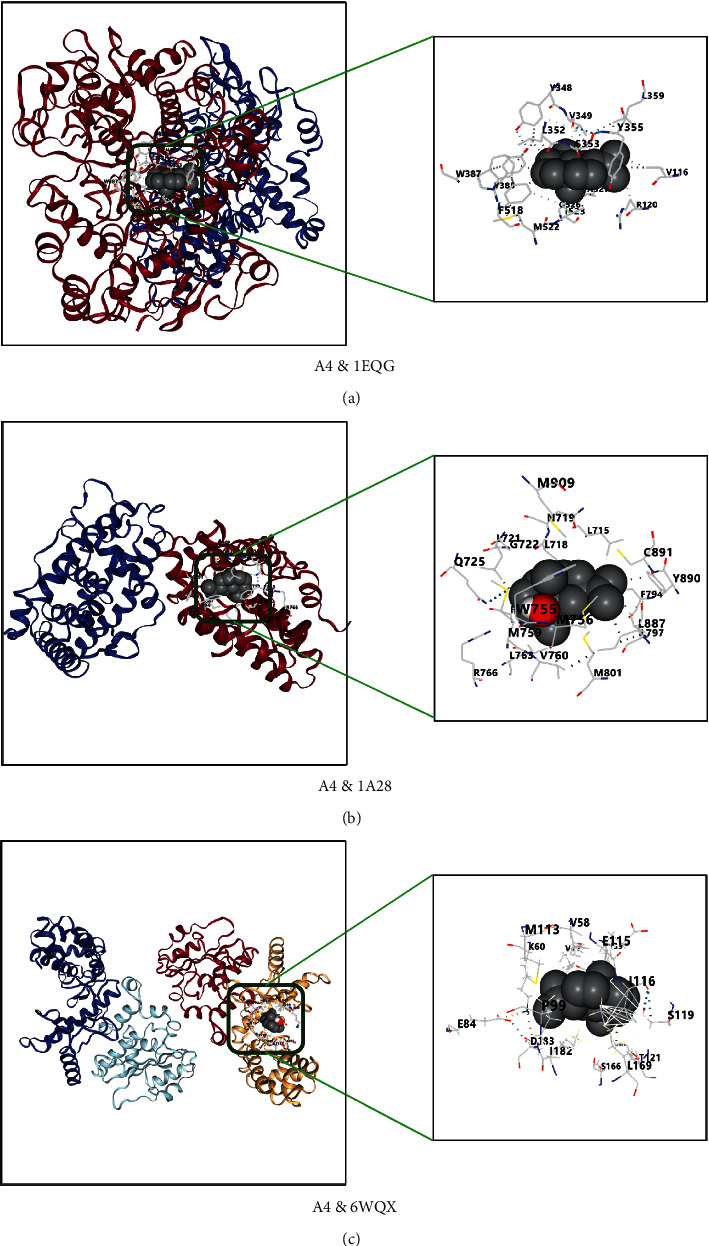
(a–c) Docking results of compound A4-neointermedeol and PTGS2 (1EQG), PGR (1A28), and TP53 (6WQX), respectively.

**Figure 7:**
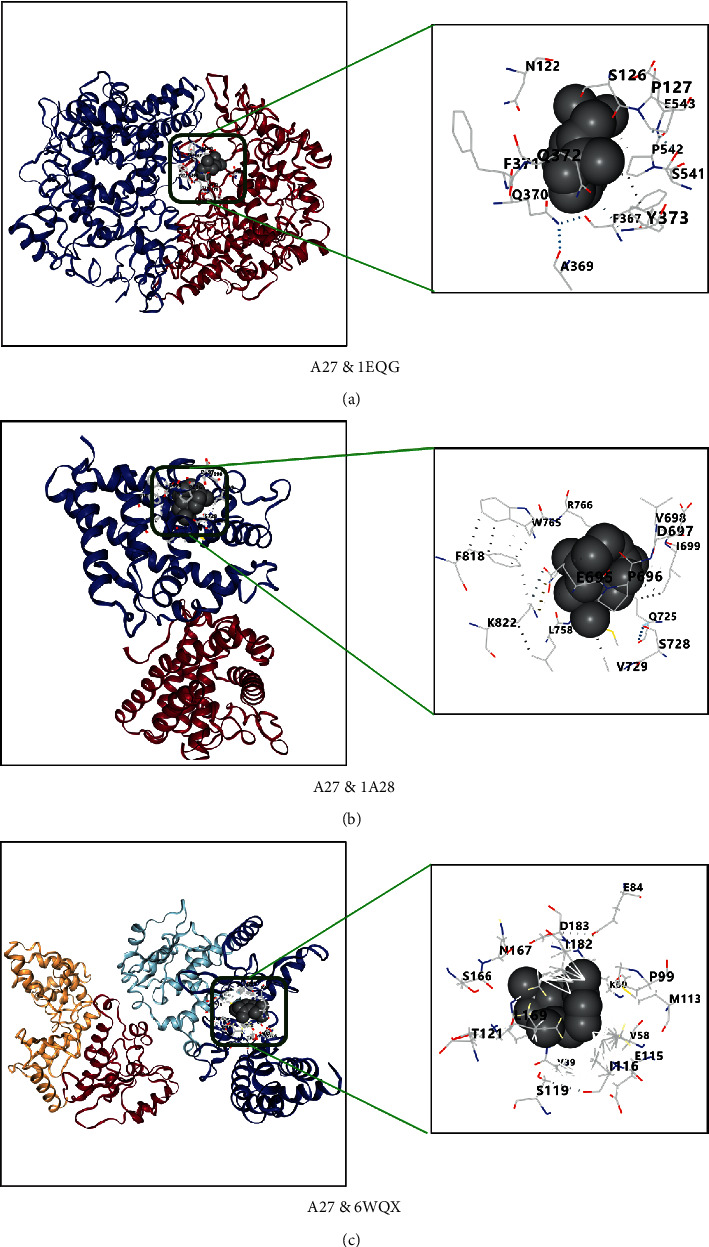
(a–c) Docking results of compound A27 *β*-caryophyllene and PTGS2 (1EQG), PGR (1A28), and TP53 (6WQX), respectively.

**Figure 8:**
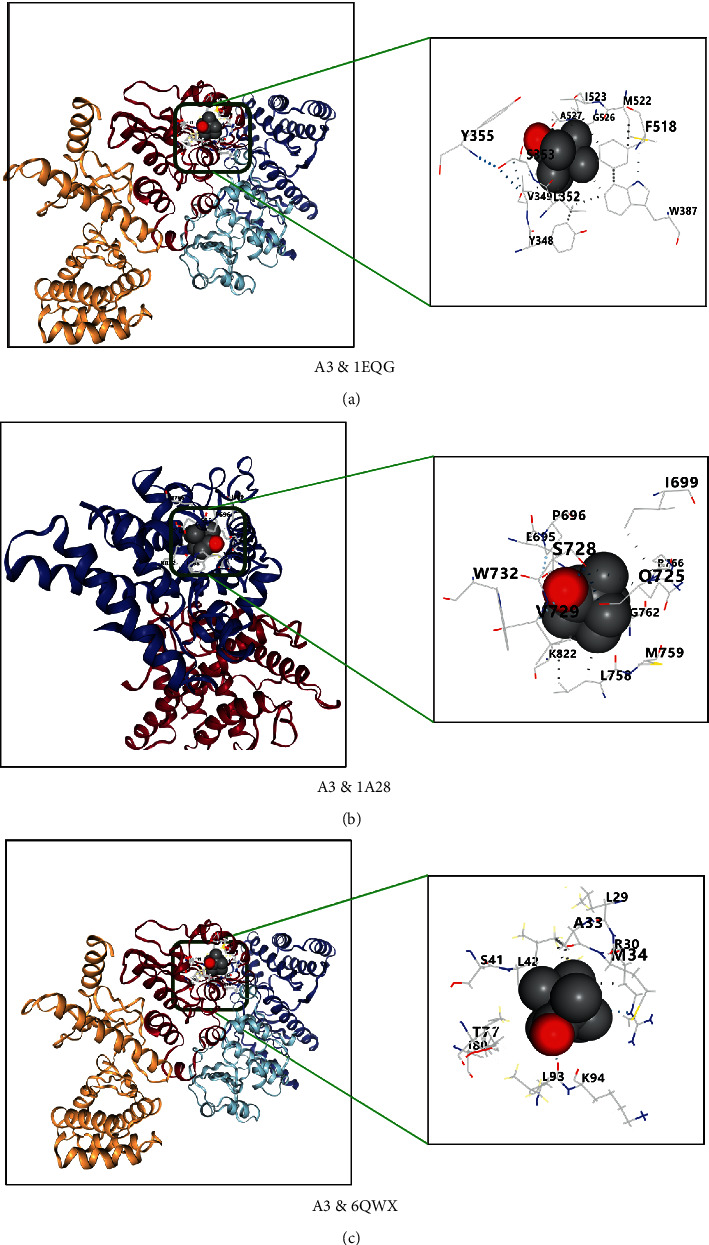
(a–c) Docking results of compound A3 terpinen-4-ol and PTGS2 (1EQG), PGR (1A28), and TP53 (6WQX), respectively.

The 10 key targets, TNF, PTGS2, IL6, IL1*β*, NR3C1, CASP3, TP53, PGR, REN, and NOS2, were docked with top 7 compounds: *β*-caryophyllene (A27),1,8-cineole (A1), terpinen-4-ol (A51), neointermedeol (A4), *α*-thujone (A11), borneol (A6), and camphor (A3). Generally, the Vina score is negative; the lower the score, the better the binding activity between ligand and protein. There will be top five Vina scores and docking cavity sizes from obtained results, which were first selected as representation [50]. The results indicated that the top 7 active compounds of AAEO had a good affinity to key targets and the RMSD of each docking target and compound was less than 2 angstroms (Tables 2 and 3). The top 3 compounds (A4-neointermedeol, A27-*β*-caryophyllene, and A3-camphor) and proteins (PTGS2, PGR, and TP53) with better binding affinities are shown in Figures 6–8.

## 4. Discussion

*Artemisia argyi*, a dried leaf of Ai Ye with multiple biological activities, is widely used to treat inflammatory diseases such as eczema, dermatitis, arthritis, allergic asthma, and colitis [52]. The pharmacological mechanisms of AAEO associated with PIS are uncertain. Our study was first used network pharmacology to discover the potential targets and regulatory molecular mechanism of AAEO on PIs treatment. As a result, we identified 54 compounds as the main active components, obtained 50 key targets, including pathway in cancer, PI3K-Akt signaling pathway, human immunodeficiency virus 1 infection, MAPK signaling pathway, and Wnt signaling pathway, demonstrated the multitarget and multipathway specialty of TCM in treating diseases.

Over 200 species of AAEO can be detected by gas chromatography-mass spectrometry (GC-MS) [34], mainly including terpenoids, ketones (aldehydes), alcohols (phenols), acids (esters), alkanes (alkenes), and other chemical constituents. In our study, *β*-caryophyllene,1,8-cineole, terpinen-4-ol, neointermedeol, *α*-thujone, borneol, and camphor had a relative content of the first 7 [14, 34–36]. 1,8-Cineole, camphor, and borneol accounted for the largest proportion of AAEO [53]. In lipopolysaccharide0 (LPS-) induced cell and mouse inflammation experiments, 1,8-cineole alleviates LPS-induced vascular endothelial cell injury, obviously inhibits the production of the inflammatory mediator, increases the release of anti-inflammatory factor IL10, and improves inflammatory symptoms [54]. Borneol significantly decreased the auricular swelling rate and pain threshold of rats by activating the p38-COX-2-PGE2 signaling pathway, which has significant analgesic and anti-inflammatory effects on PDT of acne [55]. Numerous investigations have shown various essential oils of several species containing camphor as the major component, exhibiting antimicrobial activity [56–59]. Also, the application of camphor to the skin was proved to increase local blood flow in the skin and muscle, induce both cold and warm sensations, and improve blood circulation [60]. More noteworthy is that the top three compounds of molecular docking score were neointermedeol, *β*-caryophyllene, and camphor. Neointermedeol has been shown to have antioxidant, antibacterial, and other biological activities [61, 62]. Recent studies have shown that caryophyllene can provide protection for animal cells and reduce proinflammatory mediators such as TNF-*α*, IL-1*β*, IL-6, and NF-*κ*B, thereby improving the symptoms of inflammation and oxidative stress [63, 64].

The core network calculated by MCODE had 15 targets, mostly related to inflammation, oxidative stress, and apoptosis. TNF, IL6, IL-1*β*, and PTGS2 participate in regulating inflammatory cascade reaction [65–68] and can be inhibited by inflammation in different levels by AAEO. TP53, BAX, and CASP3 regulate the apoptotic process and cell protection negatively [69, 70]. KEGG Pathways enrichment analysis is mainly involved in PI3K-Akt signaling pathway, human immunodeficiency virus 1 infection, and human T-cell leukemia virus 1 infection, MAPK signaling pathway, and Wnt signaling pathway. The study found that the PI3K-Akt pathway plays a great role in antiapoptosis and angiogenesis. The PI3K-Akt pathway phosphorylated Akt, and phosphorylated Akt first activated downstream factors Bad and Caspase-9 to play an antiapoptotic role and promote angiogenesis [71], and then phosphorylated Akt further regulated eNOS, which could promote the generation of NO [72], provide oxygen and nutrients for tissue recovery and mediate skin injury repair. Ischemic-reperfusion is recognized as the mechanism of PIs; the process includes oxidative stress, excessive release of oxygen free radicals, apoptosis, and activation of inflammatory cytokines [73]. The prediction results of our network pharmacology are mostly consistent with the progress of ischemic-reperfusion. MAPK signaling pathway is involved in the repair of PIs, increases the expression of Ras, c-Raf, MEK1, p-MEK1 protein, p-ERK1 protein, and MEK1 mRNA, promotes the proliferation of vascular endothelial cells, and accelerates microvascular regeneration and remodeling [74]. More studies have shown that the repair of pressure ulcers is highly correlated with the Wnt/*β*-catenin signaling pathway regulating the proliferation and differentiation of epithelial cells, hair follicles, and sebaceous glands [75, 76].

The above arguments verify the accuracy of this network pharmacology prediction. Besides, the docking result showed that all selected core protein and ligand have a better affinity (≤5 kcal/mol), and there were 15 docking scores ≥7 kcal/mol, indicating strong binding affinity of the compound to docking protein [77]. The RMSD of the target protein is less than 2 Å, which indicates that the docking method and parameter setting are reasonable and can be used for the next docking with components [78].

In addition, study showed that AAEO dose-dependently inhibits inflammatory mediators, such as NO, PGE2, TNF-*α*, IL-6, IL-10, IFN-*β*, and MCP-1 [79]. In the experiment of AAEO in anti-inflammatory and blood stasis animals, the effect of the lowest dose of skin administration (0.25 mL/kg) was equivalent to that oral administration of the middle dose (0.50 mL/kg) [80]. PTGS2 with the highest docking scores is a biomarker of iron death; it can inhibit the expression of inflammatory factors and apoptosis [81–83]. The future research direction can explore the way of administration, dosage, and iron death mechanism pathway.

The limitation of this study is that we have not conducted clinical or animal experiments as certification; further studies will validate the potential key targets and pathways predicted and explore the mechanism of effective components of the essential oil from *Artemisia argyi* in preventing and treating PIs by combining molecular biology and pathophysiology.

## 5. Conclusion

In conclusion, in this study, the potential targets and regulatory molecular mechanisms of AAEO in the treatment of PIs were analyzed by network pharmacology and molecular docking. In total, 54 active components and 50 potential targets were screened, mainly involving PI3K-Akt signaling pathway, pathway in cancer, PI3K-Akt signaling pathway, human immunodeficiency virus 1 infection, MAPK signaling pathway, and Wnt signaling pathway, revealing that AAEO may play a role in the treatment of PIs by reducing inflammation, inhibiting apoptosis and oxidative stress, and showing the characteristics of multitarget and multipathway. Our study provides a basis for the mechanism and further research direction of AAEO in treating PIs by combining literature research, network analysis, and molecular docking.

## Figures and Tables

**Table 1:** The basic information of potential compounds of AAEO.

No.	Molecule name	CAS	Molecular formula	Relative content (%)	References
A1	1,8-Cineole	470-82-6	C_10_H_18_O	20.91	Guan et al. [14]
A2	Caryophyllene	87-44-5	C_15_H_24_	7.50	Guan et al. [14]
A3	(-)-Camphor	76-22-2	C_10_H_16_O	5.57	Guan et al. [14]
A4	Neointermedeol	5945-72-2	C_15_H_26_O	9.65	Guan et al. [14]
A5	Caryophyllene oxide	1139-30-6	C_15_H_24_O	8.71	Guan et al. [14]
A6	(-)-Borneol	464-45-9	C_10_H_18_O	16.35	Guan et al. [14]
A7	D-Carvone	5948/4/9	C_10_H_16_O	0.25	Guan et al. [14]
A8	Bornyl acetate	76-49-3	C_12_H_20_O_2_	0.24	Guan et al. [14]
A9	4-Terpineol	562-74-3	C_10_H_18_O	5.47	Guan et al. [14]
A10	Sabinene	10408-16-9	C_10_H_16_	3.36	Guan et al. [14]
A11	*α*-Thujone	546-80-5	C_10_H_16_O	14.55	Guan et al. [14]
A12	*α*-Humulene	6753-98-6	C_15_H_24_	2.24	Guan et al. [14]
A13	Eugenol	97-53-0	C_10_H_12_O_2_	0.56	Gu et al. [36]
A14	*cis*-Carveol	1197-06-4	C_10_H_16_O	1.40	Guan et al. [14]
A15	Germacrene D	23986-74-5	C_15_H_24_	0.55	Guan et al. [14]
A16	Terpinolene	586-62-9	C_10_H_16_	0.15	Guan et al. [14]
A17	Cymene	527-84-4	C_10_H_14_	0.32	Guan et al. [14]
A18	*α*-Terpineol	10482-56-1	C_10_H_18_O	3.62	Guan et al. [14]
A19	*cis*-Carveol	1197-06-4	C_10_H_16_O	1.40	Guan et al. [14]
A20	Espatulenol	6750-60-3	C_15_H_24_O	1.51	Guan et al. [14]
A21	*γ*-Elemene	515-13-9	C_15_H_24_	0.12	Gu et al. [36]
A22	*α*-Pinene	2437-95-8	C_10_H_16_	3.84	Dai et al. [35]
A23	Piperitone	89-81-6	C_10_H_16_O	0.42	Guan et al. [14]
A24	(-)-Camphene	5794/3/6	C_10_H_16_	1.83	Dai et al. [35]
A25	Isoborneol	124-76-5	C_10_H_18_O	0.63	Dai et al. [35]
A26	*cis*-*β*-Farnesene	18794-84-8	C_15_H_24_	0.11	Dai et al. [35]
A27	*β*-Caryophyllene	87-44-5	C_15_H_24_	13.64	Guan et al. [14]
A28	*γ*-Terpinene	99-85-4	C_10_H_16_	0.24	Guan et al. [14]
A29	Spathulenol	4221-98-1	C_15_H_24_O	0.82	Dai et al. [35]
A30	Diisooctyl phthalate	27554-26-3	C_24_H_38_O_4_	0.14	Dai et al. [35]
A31	*β*-Pinene	127-91-3	C_10_H_16_	3.05	Dai et al. [35]
A32	Hexahydrofarnesyl acetone	502-69-2	C_18_H_36_O	0.77	Dai et al. [35]
A33	Tricyclene	508-32-7	C_10_H_16_	0.12	Gu et al. [36]
A34	Terpinene	99-86-5	C_10_H_16_	2.26	Gu et al. [36]
A35	Dihydroactinidiolide	15356-74-8	C_11_H_16_O_2_	0.21	Dai et al. [35]
A36	Cyclohexadiene	4221-98-1	C_10_H_16_	0.77	Dai et al. [35]
A37	n-Hexadecanoic acid	57-10-3	C_16_H_32_O_2_	0.22	Dai et al. [35]
A38	Terpinyl acetate	58206-95-4	C_12_H_20_O_2_	0.27	Dai et al. [35]
A39	Diisobutyl phthalate	84-69-5	C_16_H_22_O_4_	0.14	Dai et al. [35]
A40	Myrtenol	19894-97-4	C_10_H_16_O	0.77	Dai et al. [35]
A41	Carvacrol	499-75-2	C_10_H_14_O	0.55	Dai et al. [35]
A42	Curcumene	4176-17-4	C_15_H_22_	1.06	Dai et al. [35]
A43	*trans*-Carveol	2102-58-1	C_10_H_16_O	1.17	Dai et al. [35]
A44	(+)-Limonene	5989-27-5	C_10_H_16_	0.39	Dai et al. [35]
A45	L-Carvone	6485-40-1	C_10_H_14_O	0.11	Dai et al. [35]
A46	*cis*-*β*-Terpineol	7299-40-3	C_10_H_18_O	6.61	Dai et al. [35]
A47	*cis*-Piperitol	16721-38-3	C_10_H_18_O	3.66	Dai et al. [35]
A48	Nerolidol	7212-44-4	C_15_H_26_O	0.59	Dai et al. [35]
A49	*cis*-Jasmon	488-10-8	C_11_H_16_O	0.42	Dai et al. [35]
A50	*α*-Caryophyllene	6753-98-6	C_15_H_24_	0.37	Dai et al. [35]
A51	Terpinen-4-ol	2438-10-0	C_10_H_16_O	11.09	Dai et al. [35]
A52	(5R)-5-Isopropenyl-2-methyl-2-cyclohexen-1-ol	99-48-9	C_10_H_16_O	0.12	Dai et al. [35]
A53	Oct-1-en-3-ol	3391-86-4	C_8_H_16_O	2.57	Dai et al. [35]
A54	*α*-Phellandrene	99-86-5	C_10_H_16_	1.66	Dai et al. [35]

**Table 2:** Vina score of compound-target docking (unit: kcal/mol).

ID	A27	A1	A51	A4	A11	A6	A3
TNF	−6	−5.3	−6.6	−7.8	−5.4	−5.6	−6.8
PTGS2	−6.6	−7.1	−6.8	−7.2	−6.4	−6.2	−7.3
IL6	−6.3	−5.5	−6.7	−6.3	−5.5	−5.2	−6.9
IL1B	−6.8	−5.6	−5.5	−7	−5.3	−5.6	−5.9
NR3C1	−5.5	−5.7	−6.3	−6.1	−6.2	−5.1	−5.1
CASP3	−7	−5.9	−5.9	−6.7	−5.9	−5.4	−5.7
TP53	−7.4	−5.8	−6	−7.1	−6.1	−5.8	−7.5
PGR	−7.8	−6.7	−6.4	−7.6	−6.6	−6.2	−6.5
REN	−7.9	−6.2	−5.9	−7.8	−6.2	−6.2	−6.4
NOS2	−6.9	−5.4	−7	−7.5	−6.4	−5.3	−5.5

**Table 3:** Docking parameters.

Target	PDB ID	Ligand	Cavity size	Center			Size			RMSD
				*X*	*y*	*z*	*x*	*y*	*z*	
TNF	1D0G	A27	330	29	20	8	18	18	18	0.000
		A1	791	9	38	47	16	16	16	0.098
		A51	330	29	20	8	17	17	17	0.096
		A4	330	29	20	8	18	18	18	0.000
		A11	4587	23	55	16	35	16	28	0.585
		A6	791	9	38	47	16	16	16	0.640
		A3	1620	45	34	15	18	28	18	0.000

PTGS2	1EQG	A27	37209	48	34	189	35	35	35	0.548
		A1	1809	73	23	195	24	26	28	0,104
		A51	3589	22	28	203	26	29	24	0,103
		A4	3589	22	28	203	26	29	24	0.000
		A11	1809	73	23	195	24	26	28	0.591
		A6	1809	73	23	195	24	26	28	0.645
		A3	3589	22	28	203	26	29	24	0.000

IL6	4O9H	A27	533	−20	17	27	18	18	18	0.000
		A1	533	−20	17	27	16	16	16	0.103
		A51	533	−20	17	27	17	17	1	0.103
		A4	533	−20	17	27	18	18	18	0.000
		A11	533	−20	17	27	16	16	16	0.590
		A6	533	−20	17	27	16	16	16	0.664
		A3	533	−20	17	27	18	18	18	0.000

IL1*β*	3POK	A27	987	−15	−17	−8	18	18	18	0.000
		A1	254	21	45	26	18	18	18	0.105
		A51	987	−15	−17	−8	23	17	17	0.103
		A4	987	−15	−17	−8	18	18	18	0.000
		A11	199	−25	5	−7	16	16	16	0.592
		A6	199	−25	5	−7	16	16	16	0.646
		A3	987	−15	−17	−8	18	18	18	0.000

NR3C1	1LAT	A27	2172	31	38	81	28	32	35	0.000
		A1	2172	31	38	81	28	32	35	0.000
		A51	153	27	38	92	17	17	17	0.000
		A4	2172	31	38	81	28	32	35	0.000
		A11	2172	31	38	81	28	32	35	0.000
		A6	2172	31	38	81	28	32	35	0.000
		A3	2172	31	38	81	28	32	35	0.000

CASP3	5JFT	A27	1834	3	4	−25	18	31	18	0.000
		A1	1834	3	4	−25	17	31	17	0.104
		A51	1834	3	4	−25	17	31	17	0.103
		A4	1834	3	4	−25	18	31	18	0.000
		A11	1834	3	4	−25	17	31	17	1.153
		A6	1834	3	4	−25	16	31	16	0.646
		A3	1834	3	4	−25	16	31	16	0.592

TP53	6WQX	A27	5969	36	3	−12	26	35	31	0.000
		A1	1644	23	18	12	30	16	16	0.272
		A51	874	0	−10	49	17	17	17	0.427
		A4	5969	36	3	−12	26	35	31	0.000
		A11	1644	23	18	12	30	16	16	1.153
		A6	1644	23	18	12	30	16	16	0.586
		A3	874	0	−10	49	18	18	18	1.127

PGR	1A28	A27	617	43	34	30	18	18	18	0.000
		A1	617	43	34	30	16	16	16	0.000
		A51	631	23	5	73	17	17	17	0.000
		A4	421	23	10	60	18	18	18	0.000
		A11	617	43	34	30	17	17	17	0.000
		A6	617	43	34	30	16	16	16	0.000
		A3	617	43	34	30	16	16	16	0.000

REN	3OWN	A27	11842	9	−14	−30	35	35	33	0.000
		A1	1910	20	−1	−18	26	16	16	0.107
		A51	1834	3	4	−25	17	31	17	0.106
		A4	1910	20	−1	−18	26	18	18	0.000
		A11	1682	−10	−28	−37	17	23	17	1.156
		A6	1682	−10	−28	−37	16	23	22	0.648
		A3	11842	9	−14	−30	35	35	33	0.594

NOS2	1M7Z	A27	3650	5	33	11	30	27	30	0.000
		A1	3650	5	33	11	30	27	30	0.103
		A51	3650	5	33	11	30	27	30	0.102
		A4	3650	5	33	11	30	27	30	0.000
		A11	3650	5	33	11	30	27	30	1.152
		A6	3650	5	33	11	30	27	30	0.645
		A3	3650	5	33	11	30	27	30	0.590

## Data Availability

All data generated or analyzed during this study are included in this article.
